# What is the taxonomic status of East Asian otter species based on molecular evidence?: focus on the position of the Japanese otter holotype specimen from museum

**DOI:** 10.1080/19768354.2019.1601133

**Published:** 2019-05-02

**Authors:** Han-Chan Park, Nozomi Kurihara, Kyung Seok Kim, Mi-Sook Min, Sungyong Han, Hang Lee, Junpei Kimura

**Affiliations:** a Conservation Genome Resource Bank for Korean Wildlife, Research Institute for Veterinary Science, College of Veterinary Medicine, Seoul National University, Seoul, Republic of Korea; b The United Graduate School of Veterinary Science, Yamaguchi University, Yamaguchi, Japan; c Department of Natural Resource Ecology and Management, Iowa State University, Ames, IA, USA; d Korean Otter Research Center, Hwacheon, Republic of Korea; e Department of Anatomy and Cell Biology, College of Veterinary Medicine, Seoul National University, Seoul, Republic of Korea

**Keywords:** Cytochrome *b* gene, mitochondrial DNA, phylogenetic analysis, *Lutra lutra*, *Lutra nippon*

## Abstract

The Japanese otter (*Lutra nippon*), once inhabited in most islands of Japan, is now considered as an extinct species. Although the Japanese otter is regarded as a distinct species from the Eurasian otter (*L. lutra*), its phylogeny and taxonomic status are based on limited information on morphological and genetic data, and thus further clarification is required. Here, we assessed the phylogenetic relationship among the genus *Lutra* and taxonomic status of *L. nippon* by using the complete sequences of cytochrome *b* gene of its holotype. The present phylogenic trees supported that the genus *Lutra* specimens largely formed monophyletic group, with *L. sumatrana* as a basal to other *Lutra* species. Within *Lutra* species, *L. nippon* was distantly related with *L. lutra*. The European otter population of *L. l. lutra* were clustered together with its subspecies, *L. l. chinensis* rather than the same subspecies, Korean otter population. The discrepancy between the genetic data and traditional taxonomy justifies the necessity of reexamination of the current subspecific classification system of Eurasian otters. Level of genetic divergence between the holotype of *L. nippon* and *L. lutra* was two to three-fold lower than those among the other sister species of the Lutrinae. Based on the level of divergence between the *L. nippon* and *L. lutra*, and insufficient evidence of morphological difference between them, it is suggested that designation of Japanese otter as a separate species from L. *lutra* will be reconsidered.

## Introduction

The Japanese otter (*Lutra nippon*) once inhabited most of the main Japanese islands except Hokkaido (Sasaki 1995). However, due to a rapid decline in its population, it has not been sighted since the 1980s and was officially declared extinct by the Japanese government on 29 August 2012 (Kyodo News). Since the 1980s, there has been increased attention to this species (Yamamoto and Ando 2011) and it has become the subject of several morphological and genetic studies. These studies largely concluded that *L. nippon* is a distinct species separate from the Eurasian otter *Lutra lutra* (Imaizumi and Yoshiyuki 1989; Suzuki et al. 1996; Endo et al. 2000). However, the taxonomic status of *L. nippon* still remains uncertain because the conclusion is derived from limited morphological and genetic data.

According to Wozencraft (2005), the genus *Lutra* includes three species: the Eurasian otter, *L. lutra*; the hairy-nosed otter, *L. sumatrana*; and the Japanese otter, *L. nippon*. *L. sumatrana* and *L. nippon* have limited distributions in southeast Asia and the Japanese islands, respectively. On the contrary, *L. lutra* is widely distributed across the Eurasian continent including south of the tundra line and in north Africa, and is divided into seven subspecies (Pocock 1941; Roos et al. 2015): *L. l. lutra* is distributed in Eurasia from England to the Korean peninsula, excluding India, southeast Asia, and southern China; *L. l. chinensis* inhabits the southern part of China and Kimmen Island of Taiwan; and the remaining five subspecies (*L. l. barang*, *L. l. nair*, *L. l. monticola*, *L. l. kutab*, and *L. l. aurobrunnea*) are distributed in the southern part of Asia. *L. nippon* was traditionally identified as a subspecies of *L. lutra*, *L. l. whiteleyi*, which is synonymous with *L. l. lutra*, based on morphological features of its skin and skull (Gray 1867; Imaizumi 1949). Conversely, Imaizumi and Yoshiyuki (1989) suggested that *L. nippon* was a distinct species, based on skulls from the Shikoku, Honshu, and Hokkaido areas, which were morphologically distinct from the skulls of *L. lutra*, including *L. l. whiteleyi*. Likewise, Suzuki et al. (1996) found *L. nippon* to be a distinct species from *L. lutra* based on the genetic difference of 3.6% in the partial sequences of its cytochrome *b* gene (224 bp). The length of analyzed sequence in their study was, however, too short to define a species. Furthermore, the sample size in their study was not sufficient to compare inter- and intraspecific variation among the genus *Lutra*. Therefore, Roos et al. (2015) concluded that the taxonomic position of *L. nippon* remains uncertain requiring further studies and cannot view it as a separate species from *L. lutra*. Regarding morphological differences, Lau et al. (2016) identified that *L. lutra* from South Korea is sexually dimorphic. Therefore, sexual dimorphism in *L. nippon* is a possible confounding effect and thus interpretation should be cautioned when using morphology to evaluate their taxonomy. Waku et al. (2016) analyzed the phylogenetic relationship among *Lutra spp.*, including *L. nippon*, using mitochondrial genome sequences (14,740 bp) and consequently divided the Japanese populations into two lineages: *L. lutra* and another *Lutra* species or subspecies. Additionally, Waku et al. (2016) identified two lineages of Eurasian otter (*L. lutra*) in East Asia; one lineage comprising of Chinese otter (*L. l. chinensis*) and another comprising of Eurasian otter (*L. l. lutra*) from South Korea and Sakhalin, Russia. However, limited sampling of the Eurasian otter range in East Asia led to phylogenetic relationships among East Asian otter populations an uncertain state. Koh et al. (2004) concluded that partial mitochondrial DNA sequence of Korean otter was distinct from those of European otters, but authors provided limited information on relationships of Eurasian otter populations. Therefore, the phylogenetic relationship of Eurasian otters at species and subspecies level in East Asia still remains unclear and the taxonomic status of *L. nippon* remains controversial.

Genetic markers based on mitochondrial DNA, such as the cytochrome *b* gene, hypervariable portion of the control region (D-loop), and cytochrome c oxidase I, have been used for phylogenetic and population genetic analysis for most mammalian taxa. Specifically, the cytochrome *b* gene sequences have been used to investigate relationships among mammalian taxa at a family-subspecific level (Ledje and Arnason 1996; Johns and Avise 1998; Koepfli and Wayne 1998; Bradley and Baker 2001; Kurose et al. 2008; Koepfli, Deere, et al. 2008; Koepfli, Kanchanasaka, et al. 2008). Hence, the objective of this study is to determine the molecular phylogeny of *L. nippon* using the cytochrome *b* gene and to clarify its taxonomic status. Only few Japanese otter specimens have a reliable information on locality, and therefore we focused on the relationship among the holotypes of *L. nippon* (Imaizumi and Yoshiyuki 1989), *L. lutra*, and *L. sumatrana*. We also investigated the phylogenetic relationship of Eurasian otters at subspecific level.

## Materials and methods

Samples examined in this study and sequence data from GenBank are summarized in Table 1. The holotype of *L. nippon* (Imaizumi and Yoshiyuki 1989) was employed in this study. This holotype was collected in 1972 from the Nenokubi seaside of Kochi Prefecture in Japan where its skeleton and mounted skin are preserved in the National Museum of Nature and Science, Tokyo, Japan.

**Table 1.** Sample and DNA sequence information used in this study.

Species		ID	Locality	GenBank sequence ID
Scientific name	Common name			
*Lutra nippon*	Japanese otter	JP1	Nenocubi seaside, Kochi, Japan	LC006975
		JP2	Hatagun, Kochi, Japan	^a^LC050126
*L. l. lutra*	Eurasian otter of South Korea	KO1	Busan, South Korea	KU953401
		KO2	Gurye, South Korea	KU953402
		KO3	Gangleung, South Korea	KU953403
		KO4	Yeosu, South Korea	KU953404
		KO5	South Korea	^b^FJ236015
		KO6	South Korea	^c^EF672696
	Eurasian otter of Europe	EU1	Norway	^d^AF057124
		EU2	Portugal	^e^EF689067
*L. l. chinensis*	Eurasian otter of China	CH1	China	^a^LC049952
*L. l. spp*		CH2	China	^a^LC049378
*L. l. spp*		CH3	China	^a^LC049377
*L. sumatra*	Hairy-nosed otter		Vietnam	^f^EF472347
*Aonyx capensis*	African clawless otter		South Africa	^d^AF057118
*Aonyx cinereus*	Oriental small-clawed otter		–	^d^AF057119
*Lutrogale perspicillata*	Smooth-coated otter		Thailand	^f^EF472348
*Lontra felina*	Marine otter		Chile	^d^AF057122
*Lontra longicaudis*	Neotropical otter		–	^d^AF057123
*Lontra canadensis*	North American river otter		USA	^d^AF057121
*Taxidea taxus*	American badger		USA	^d^AF057132

^a^Waku et al. (2016).

^b^Jang et al. (2009).

^c^Ki et al. (2010).

^d^Koepfli and Wayne (1998).

^e^Fernandes et al. (2008).

^f^Koepfli, Kanchanasaka, et al. (2008).

Six *L. l. lutra* specimens from the Korean peninsula were also examined to assess the possible sequence variation among *L. l. lutra* individuals. Korean otter specimens were collected from several areas in South Korea and they were obtained from a variety of sources, including individuals that were road killed, caught in a fishing net or illegal trap, or that had been rescued as cubs but subsequently died. These tissue samples had been preserved in the Conservation Genome Resource Bank for Korean Wildlife (CGRB) and Association of Korean Otter Conservation (AKOC) with proper permits from the Cultural Heritage Administration (CHA) of South Korean government because Korean otter is designated as a natural monument species by the CHA. The sequence data of complete cytochrome *b* genes of *L. l. lutra* from Europe and South Korea, *L. l. chinensis*, *L. nippon*, *L. sumatrana*, *Aonyx capensis*, *A. cinereus*, *Lontra felina*, *Lontra longicaudis*, *Lontra canadensis* (all subfamily Lutrinae), and *Taxidea taxus* (family Mustelidae) were cited from GenBank as reference data.

Total DNA from the holotype of *L. nippon* was extracted from dried costal cartilage that had been preserved in the National Museum of Nature and Science, Tokyo. The cartilage was washed in 99% ethanol after treatment with a TE buffer. The cartilage pieces were cut into 0.5–1.0 cm and then decalcified with EDTA (pH 8.0) at room temperature for 5 days. DNA was extracted using an Ultra Clean™ Tissue DNA Isolation Kit (MO BIO Laboratories Inc.) following the manufacturer’s protocol. All procedure of DNA extraction from the holotype specimen was performed in the clean bench for preventing contamination.

Polymerase chain reaction (PCR) amplification of the cytochrome *b* gene of the holotype of *L. nippon* was performed in a 10-µL reaction volume containing the following reagents: 10× Ex *Taq* Buffer (Takara Bio Inc.), 0.5 mM of each dNTP mix (Takara Bio Inc.), 2 µM forward and reverse primers (Supplement A, B), 0.5 U Ex *Taq* (Takara Bio Inc.), and 1.0 µL template DNA. Amplification was conducted for a total of 46 cycles using the 14 primers designed in the present study (Supplement A, B). The conditions for the initial 10 cycles were as follows: 94°C for 30 s, 45°C for 20 s, and 72°C for 20 s; and the conditions for the remaining 36 cycles were: 94°C for 30 s, 55°C for 20 s, and 72°C for 20 s. Each of the partial cytochrome *b* sequences of amplicons that were amplified by a combination of 14 primers was analyzed using an IBM 3130 sequencer analyzer (Applied Biosystems™), and those sequences were aligned and assembled using Geneious Pro v5.3 to obtain the complete cytochrome *b* sequence (1140 bp) from the holotype of *Lutra nippon*.

Genomic DNA of *L. l. lutra* from Korea was extracted from the tissue using a DNA extraction kit (Blood & Tissue Kit, QiagenTM) according to the manufacturer’s manual. PCR amplification of the complete cytochrome *b* gene from the Korean population of *L. l. lutra* was carried out under the following conditions: one cycle of 94°C for 4 min, 35 cycles of 94°C for 30 s, 40°C for 60 s, 72°C for 90 s, and a final cycle of 72°C for 5 min. Each 30-µL reaction volume contained 10× PCR buffer (iNtRON Biotechnology, Inc.), 0.2 mM of each dNTP mix (iNtRON Biotechnology,Inc.), 0.5 µM forward (L14724: CGA AGC TTG ATA TGA AAA ACC ATC GTT G) and reverse (H15915: AAC TGC AGT CAT CTC CGG TTT ACA AGA C) primers (Collura et al. 1996), 1U *i*-Star*Taq* (iNtRON Biotechnology, Inc.), and 1.5 µL template DNA (30 ng). Six complete cytochrome *b* sequences (1140 bp) were analyzed using an ABI3730 XL sequencer analyzer (Applied Biosystems™). Sequences were aligned using Geneious Pro v5.3 (Kearse et al. 2012).

The pairwise genetic distance among those sequences was calculated by using PAUP4.0 based on Kimura 2 Parameter (Kimura 1980). Jmodeltest2.1.8 was used to find the best-fit substitution model of sequence evolution for constructing phylogenetic trees (Posada 2008). A maximum likelihood (ML) tree was reconstructed using PAUP4.0 (Swofford 2001), with an application of 1000 pseudoreplicates of this ML tree to obtain bootstrap support values. The Bayesian inference (BI) tree was obtained using MrBayes 3.2.3 (Ronquist et al. 2012). BI employed four simultaneous Monte Carlo Markov chains (one cold and three heated) with 1,000,000 generations and sampled every 500 generations. The first 25% of the data points were discarded as burn-in. The consensus trees from both ML and BI were illustrated using FigTree v 1.4.3 (http://tree.bio.ed.ac.uk/software/figtree/).

## Results and discussion

**Figure 1.**
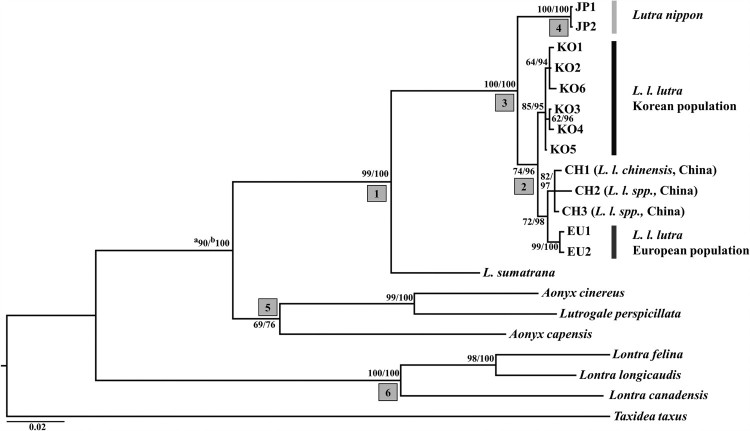
Phylogenetic tree reconstructed by Bayesian method using MrBayes 3.1.3, and this Bayesian tree has same topology with maximum likelihood tree (-Ln likelihood = 4427.196) reconstructed by PAUP4.0. (a) Bootstrap support values (%) obtained from 1000 pseudoreplicates of maximum likelihood tree. (b) Posterior probabilities (%) from Bayesian method.

The phylogenic relationship among Lutrinae species was constructed using the ML and Bayesian methods under GTR + G substitution model, which was selected from Jmodeltest 2.1.8, based on their Akaike information criterion (AIC = 8952.42) value (Figure 1). The phylogenic tree supported that the genus *Lutra* specimens largely formed a monophyletic group, with *L. sumatrana* as a basal to other *Lutra* species (Figure 1, node 1). In the Chinese otter group, CH2 and CH3 were clustered with *L. l. chinensis* (CH1), so they were presumed to be the same subspecies, *L. l. chinensis*. Within *Lutra* species, the European otter population of *L. l. lutra* were clustered together with its subspecies, *L. l. chinensis* (or *L. l. spp*) from China rather than the same subspecies, Korean otter population (Figure 1, node 2). The results of the molecular phylogenetic analysis in this study are not in agreement with the traditional subspecific taxonomic system of *L. lutra,* in which Korean otters and European otters are classified as the same subspecies (*L. l. lutra*), and otters in Southern part of China are regarded as a distinguished subspecies, *L. l. chinensis*. However, the results of this study identified haplotypes of Korean otter population as a monophyletic group distinct from the European and Chinese otter populations (Figure 1, node 2) implying that Korean otters had been branched out earlier than the divergence between *L. l. chinensis* and European otters of *L. l. lutra*. The discrepancy between the genetic data and traditional taxonomy justifies the necessity of reexamination of the current subspecific classification system of Eurasian otters.

It is notable that a clade clustering the *L. nippon* and *L. lutra* specimens was strongly supported by both phylogenetic trees, with a bootstrap value of 100% in ML trees and a posterior probability of 100% in BI tree (Figure 1, node 3). The phylogenetic relationship showed that the holotype of *L. nippon* (JP1) and another Japanese otter (JP2) identified by Waku et al. (2016) were a member of the genus *Lutra* (Figure 1, node 1, 4). Furthermore, the holotype of *L. nippon* formed a monophyletic group with *L. lutra* although it was reported that they were isolated from two other *Lutra* species, *L. lutra* and *L. sumatrana* (Imaizumi and Yoshiyuki 1989; Suzuki et al. 1996). The phylogenetic relationship shown in this study was consistent with that of Waku et al. (2016), who used part of the 14,740 bp mitochondrial genome sequences for comparison. Waku et al. (2016) identified two lineages that one belongs to *L. lutra* and the other is an old Japanese lineage, and thus they were regarded as either a new *Lutra* species or a subspecies of *L. lutra*. Since complete cytochrome *b* sequences of the old Japanese lineage (JP2) in Waku et al. (2016) is identical with that of the holotype of *L. nippon*, the lineage is considered to represent *L. nippon* (Figure 1. node 4). Pairwise genetic distances of the cytochrome *b* gene among the holotype of *L. nippon*, *L. lutra*, including *L. l. lutra* and *L. l. chinensis*, and the other seven species of Lutrinae are shown in Table 2. In spite of the small sample size, our findings based on genetic distance support the distinction of *L. nippon* from *L. lutra*. The holotype of *L. nippon* had genetic distances of 2.4–2.8% and 2.8–3.3% from *L. l. lutra* and *L. l. chinensis* (or *L. l. spp*) from China, respectively, whereas the distance between the two *Lutra* subspecies, *L. l. lutra* and *L. l. chinensis*, was only 0.8–1.4%. Korean and European populations of *L. l. lutra*, respectively located at the east and west extreme of Eurasian continent, had a genetic distance of 0.9–1.2% between them despite long geographic distance. It is thus unlikely that *L. nippon* is part of the variation within *L. lutra*. Johns and Avise (1998) concluded that the genetic distance of the cytochrome *b* gene among mammalian sister species generally ranged from 2% to 24%. According to their index, the genetic distance between the holotype of *L. nippon* and *L. lutra* (*L. l. lutra *+ *L. l. chinensis*, 2.4–3.3%) found in this study suggests that they differ at a marginal level. The genetic divergence between the holotype of *L. nippon* and *L. lutra* was two to three-fold smaller than those between the sister species in the subfamily Lutrinae (6.7–7.2% between *L. lutra* and *L. sumatrana*, 8.1–11.6% among *A. capensis*, *A. cinereus*, and *Lutrogale perspicillata* (Figure 1, node 5), and 5.8–11% among *L. felina*, *L. longicaudis*, and *L. canadensis* (Figure 1, node 6)). Therefore, it is suggested that the holotype of *L. nippon* diverged from *L. lutra* at a boundary point between a subspecies and a species of the genus *Lutra*.

**Table 2.** Pairwise genetic distance based on cytochrome *b* gene (1140 bp) sequence variance.

		1	2	3	4	5	6	7	8	9	10	11	12	13	14	15	16	17	18	19	20	21
1	JP1 (*Lutra nippon*)																					
2	JP2 (*L. nippon*)	.000																				
3	KO1 (*L. l. lutra*)	.026	.026																			
4	KO2 (*L. l. lutra*)	.025	.025	.001																		
5	KO3 (*L. l. lutra*)	.025	.025	.003	.002																	
6	KO4 (*L. l. lutra*)	.026	.026	.004	.003	.001																
7	KO5 (*L. l. lutra*)	.024	.024	.002	.001	.001	.002															
8	KO6 (*L. l. lutra*)	.027	.027	.003	.002	.004	.004	.003														
9	CH1 (*L. l. chinensis*)	.029	.029	.010	.009	.009	.010	.008	.011													
10	CH2 (*L. l. spp.*)	.033	.033	.013	.012	.012	.013	.012	.014	.007												
11	CH3 (*L. l. spp.*)	.028	.028	.009	.008	.008	.009	.007	.010	.003	.006											
12	EU1 (*L. l. lutra*)	.028	.028	.011	.010	.010	.011	.009	.012	.008	.012	.007										
13	EU2 (*L. l. lutra*)	.028	.028	.011	.010	.010	.011	.009	.012	.008	.012	.007	.002									
14	*L. sumatrana*	.072	.072	.069	.068	.066	.067	.067	.068	.070	.069	.067	.067	.067								
15	*Aonyx capensis*	.132	.132	.124	.123	.125	.126	.124	.123	.127	.126	.124	.126	.126	.121							
16	*Aonyx cinereus*	.129	.129	.124	.123	.126	.124	.124	.123	.126	.127	.122	.128	.128	.115	.116						
17	*Lutrogale perspicillata*	.118	.118	.109	.110	.112	.111	.111	.108	.108	.111	.107	.112	.112	.107	.113	.081					
18	*Lontra felina*	.165	.165	.158	.157	.160	.158	.158	.157	.160	.163	.161	.155	.155	.161	.167	.165	.165				
19	*Lontra longicaudis*	.170	.170	.164	.163	.165	.164	.164	.163	.165	.169	.164	.161	.161	.162	.172	.163	.162	.058			
20	*Lontra canadensis*	.165	.165	.161	.162	.162	.161	.161	.164	.169	.170	.168	.164	.164	.173	.181	.177	.177	.110	.102		
21	*Taxidea taxus*	.164	.164	.163	.162	.162	.163	.161	.162	.166	.162	.162	.165	.165	.158	.166	.185	.167	.185	.192	.195	

Despite the geographic proximity of Korea to Japan than Europe, Korean otter populations are more closely related to those of Europe than to Japanese populations. A similar pattern has been reported in other mammalian species such as the Siberian flying squirrel (*Pteromys volans*), the Asiatic black bear (*Ursus thibetanus*), and the raccoon dog (*Nyctereutes procyonoides*) (Lee et al. 2008; Kim et al. 2011, 2013). The isolation and differentiation of *L. nippon* population are assumed owing to the geographic isolation of the Japanese islands from the Eurasian continent by the sea. Over time, vicariance such as the geographic isolation of *L. nippon* from *L. lutra* in the Eurasian continent may have led to its speciation. Indeed, *L. nippon* also exhibits a certain level of morphological divergence from *L. lutra*. Imaizumi and Yoshiyuki (1989) described *L. nippon* as being generally similar to *L. lutra* but with certain morphological differences, with, in the former species, a larger skull with a longer facial portion, a relatively small inner lobe of P4 (protocone), a longer tail, and a naked and larger rhinalium. Endo et al. (2000) also outlined some obvious differences in skull shape between *L. nippon* and the continental *L. lutra* populations using multivariate analyses. Whereas, Waku et al. (2016) identified two lineages of otters from Japan – *L. lutra* and another *Lutra* species or subspecies – the latter being considered as *L. nippon* (Imaizumi and Yoshiyuki 1989). If *L. nippon* and *L. lutra* had occurred sympatrically in Japan, the evidence of genetic difference between the holotype of *L. nippon* and *L. lutra* found in our study could be explained by the existence of reproductive barriers between them. However, there is no evidence of sympatry between *L. nippon* and *L. lutra*. Both the holotype of *L. nippon* in this study and the specimen examined by Waku et al. (2016), which was considered to be the *L. nippon* lineage, were obtained from the Kochi Prefecture, whereas the specimen regarded as *L. lutra* in Waku et al. (2016) was from Jogashima, Kanagawa Prefecture. Furthermore, Waku et al. (2016) stated that *L. lutra* from Jogashima may have been brought there artificially. Further clarification of the taxonomic status of *L. nippon* requires the inclusion of more specimens from the Jogashima region. Although *L. nippon* has diverged from *L. lutra* at certain genetic and morphological levels, there seems insufficient evidence yet to categorize it as a distinct species.

Based on the level of divergence between the *L. nippon* and *L. lutra*, and limited evidence of morphological difference between them, it is, therefore, suggested that designation of Japanese otter as a separate species from *L. lutra* will be reconsidered until comprehensive and robust evidence supporting independent specific status of *L. nippon* are discovered. In addition, taxonomic classification of a regionally extirpated population as a separate species without concrete scientific evidence would not be desirable because it may preclude the potential restoration or reintroduction planning or discussion of the species into the historical range in the future.

## Supplementary Material

Supplemental Material

Supplemental Material

## References

[CIT0001] Bradley RD, Baker RJ. 2001 A test of the genetic species concept: cytochrome-*b* sequences and mammals. J Mammal. 82:960–973. doi: 10.1644/1545-1542(2001)082<0960:ATOTGS>2.0.CO;2 PMC2771874 19890476

[CIT0002] Collura RV, Auerbach MR, Stewart C-B. 1996 A quick, direct method that can differentiate expressed mitochondrial genes from their nuclear pseudogenes. Curr Biol. 6:1337–1339. doi: 10.1016/S0960-9822(02)70720-3 8939570

[CIT0003] Endo H, Ye X, Kogiku H. 2000 Osteometrical study of the Japanese otter (*Lutra nippon*) from Ehime and Kochi prefectures. Mem Natl Sci Mus (Tokyo). 33:195–201.

[CIT0004] Fernandes CA., Ginja C., Pereira I., Tenreiro R., Bruford MW., Santos-Reis M. 2008 Species-specific mitochondrial DNA markers for identification of non-invasive samples from sympatric carnivores in the Iberian Peninsula. Conserv Genet. 9:681. doi:10.1007/s10592-007-9364-5.

[CIT0005] Gray JE. 1867 Notice of Lutronectes whiteleyi, an otter from Japan. P Zool Soc Lond. 35:180–182.

[CIT0006] Imaizumi Y. 1949 The natural history of Japanese mammals. Tokyo: Yoyo shobo p. 348.

[CIT0007] Imaizumi Y, Yoshiyuki M. 1989 Taxonomic status of the Japanese otter (Carnivora, Mustelidae), with a description of a new species. Bull Natl Sci Mus Ser A Zool. 15:177–188.

[CIT0008] Jang KH, Ryu SH, Hwang UW. 2009 Mitochondrial genome of the Eurasian otter *Lutra lutra* (Mammalia, Carnivora, Mustelidae). Genes Genom. 31:19–27. doi: 10.1007/BF03191134

[CIT0009] Johns GC, Avise JC. 1998 A comparative summary of genetic distances in the vertebrates from the mitochondrial cytochrome *b* gene. Mol Biol Evol. 15:1481–1490. doi: 10.1093/oxfordjournals.molbev.a025875 12572611

[CIT0010] Kearse M, Moir R, Wilson A, Stones-Havas S, Cheung M, Sturrock S, Buxton S, Cooper A, Markowitz S, Duran C, et al. 2012 Geneious Basic: an integrated and extendable desktop software platform for the organization and analysis of sequence data. Bioinformatics. 28:1647–1649. doi: 10.1093/bioinformatics/bts199 22543367 PMC3371832

[CIT0011] Ki JS, Hwang DS, Park TJ, Han SH, Lee JS. 2010 A comparative analysis of the complete mitochondrial genome of the Eurasian otter *Lutra lutra* (Carnivora; Mustelidae). Mol Biol Rep. 37:1943–1955. doi: 10.1007/s11033-009-9641-0 19757186

[CIT0012] Kim YK, Hong YJ, Min MS, Kim KS, Kim YJ, Voloshina I, Myslenkov A, Smith GJ, Cuong ND, Tho HH, et al. 2011 Genetic status of Asiatic black bear (*Ursus thibetanus*) reintroduced into South Korea based on mitochondrial DNA and microsatellite loci analysis. J Hered. 102:165–174. doi: 10.1093/jhered/esq121 21325020

[CIT0013] Kim SI, Park S-K, Lee H, Oshida T, Kimura J, Kim YJ, Nguyen ST, Sashika M, Min MS. 2013 Phylogeography of Korean raccoon dogs: implications of peripheral isolation of a forest mammal in East Asia. J Zool. 290:225–235. doi: 10.1111/jzo.12031

[CIT0014] Kimura M. 1980 A simple method for estimating evolutionary rates of base substitutions through comparative studies of nucleotide sequences. J Mol Evol. 16:111–120. doi: 10.1007/BF01731581 7463489

[CIT0015] Koepfli KP, Deere KA, Slater GJ, Begg C, Begg K, Grassman L, Lucherini M, Veron G, Wayne RK. 2008 Multigene phylogeny of the Mustelidae: Resolving relationships, tempo and biogeographic history of a mammalian adaptive radiation. BMC Biol. 6:10. doi: 10.1186/1741-7007-6-10 18275614 PMC2276185

[CIT0016] Koepfli K-P, Kanchanasaka B, Sasaki H, Jacques HL, Louie KDY, Hoai T, Dang NX, Geffen E, Gutleb A, Han S-Y, et al. 2008 Establishing the foundation for an applied molecular taxonomy of otters in Southeast Asia. Conserv Genet. 9:1589–1604. doi: 10.1007/s10592-007-9498-5

[CIT0017] Koepfli KP, Wayne RK. 1998 Phylogenetic relationships of otters (Carnivora: Mustelidae) based on mitochondrial cytochrome *b* sequences. J Zool Lond. 246:401–416. doi: 10.1111/j.1469-7998.1998.tb00172.x

[CIT0018] Koh HS, Yoo MH, Lee BG, Park JG. 2004 Molecular DNA systematic analyses of East Asian mammals: sequence variation of cytochrome *b* gene and control region of mitochondrial DNA of common otter, *Lutra lutra lutra* L. (Mammalia, Carnivora) from Korea. Korean J Biol Sci. 8:231–233. doi: 10.1080/12265071.2004.9647755

[CIT0019] Kurose N, Abramov AV, Masuda R. 2008 Molecular phylogeny and taxonomy of the genus *Mustela* (Mustelidae, Carnivora), inferred from mitochondrial DNA sequences: new perspectives on phylogenetic status of the back-striped weasel and American mink. Mamm Study. 33:25–33. doi: 10.3106/1348-6160(2008)33[25:MPATOT]2.0.CO;2

[CIT0020] Kyodo News 2012 Aug 29 Japanese river otter declared extinct. The Japan Times. p. 1.

[CIT0021] Lau ACC, Asahara M, Han SY, Kimura J. 2016 Sexual dimorphism of the Eurasian otter (*Lutra lutra*) in South Korea: Craniodental geometric morphology. J Vet Med Sci. 78:1007–1011. doi: 10.1292/jvms.16-0018 26983684 PMC4937134

[CIT0022] Ledje C, Arnason U. 1996 Phlyogenetic analyses of complete cytochrome *b* genes of the order Carnivora with particular emphasis on the caniformia. J Mol Evol. 42:135–144. doi: 10.1007/BF02198839 8919865

[CIT0023] Lee MY, Park SK, Hong YJ, Kim YJ, Voloshina I, Myslenkov A, Saveljev AP, Choi TY, Piao RJ, An JH, et al. 2008 Mitochondrial genetic diversity and phylogenetic relationships of Siberian flying squirrel (*Pteromys volans*) populations. Animal Cells Syst. 12:269–277. doi: 10.1080/19768354.2008.9647182

[CIT0024] Pocock RI. 1941 The fauna of British India including Ceylon and Burma. Vol. II. London: Taylor and Francis; p. 503.

[CIT0025] Posada D. 2008 jModelTest: Phylogenetic Model Averaging. Molecular Biology and Evolution. 25(7):1253–1256. doi: 10.1093/molbev/msn083 18397919

[CIT0026] Ronquist F, Teslenko M, Mark P, Ayres DL, Darling A, Höhna S, Larget B, Liu L, Suchard MA, Huelsenbeck JP. 2012 Mrbayes 3.2: Efficient Bayesian phylogenetic inference and model choice across a large model space. Syst Biol. 61:539–542. doi: 10.1093/sysbio/sys029 22357727 PMC3329765

[CIT0027] Roos A, Loy A, de Silva P, Hajkova P, Zemanová B. 2015 *Lutra lutra*. The IUCN Red List of Threatened Species 2015.

[CIT0028] Sasaki H. 1995 History of river otters in Japan. In: Proceedings of Korea-Japan otter symposium. p. 16–17.

[CIT0029] Suzuki T, Yuasa H, Machida Y. 1996 Phylogenetic position of the Japanese river otter *Lutra nippon* inferred from the nucleotide sequence of 224 bp of the mitochondrial cytochrome *b gene*. Zool Sci. 13:621–626. doi: 10.2108/zsj.13.621 8940916

[CIT0030] Swofford DL. 2001 PAUP*. Phylogenetic analysis using parsimony (*and other methods). Version 4. Sunderland (MA): Sinauer Associates.

[CIT0031] Waku D, Segawa T, Yonezawa T, Akiyoshi A, Ishige T, Ueda M, Ogawa H, Sasaki H, Ando M, Kohno N, et al. 2016 Evaluating the phylogenetic status of the extinct Japanese otter in the basis of mitochondrial genome analysis. Plos One. DOI:10.1371/journal.pone.0149341. PMC4777564 26938434

[CIT0032] Wozencraft WC. 2005 Mammal species of the world: a taxonomic and geographic reference. 3rd ed Baltimore (MD): Johns Hopkins University Press; p. 532–628.

[CIT0033] Yamamoto K, Ando M. 2011 Trends in otter-related newspaper articles in Japan over 135 Years. Proceedings of XIth International Otter Colloquium, IUCN Otter Specialist Group. Bull. 28:31–35.

