# Supplementary material for: What is the taxonomic status of East Asian otter species based on molecular evidence?: focus on the position of the Japanese otter holotype specimen from museum
Source: Anim Cells Syst (Seoul). 2019 May 2;23(3):228–34. doi: 10.1080/19768354.2019.1601133 (PMC6567078; doi:10.1080/19768354.2019.1601133)
Supplement: Supplemental Material [file TACS_A_1601133_SM5944.docx]

Supplement B. Primer list for PCR amplification of mitochondrial cytochrome *b* DNA from the museum specimen of Japanese otter

| Primer | |  |
| --- | --- | --- |
| Name | Sequence |  |
| CBF1-1LL | GTATGTCATCATTATTCCTACATGG | Forward |
| CBR375LL | TGCTATGGTTGCGAATAGTA | Reverse |
| CBF198LL | CGCACACATTTGCCGAGACG | Forward |
| CBR461LL | GGGATGGCTGATAGTAAGTT | Reverse |
| CBF256LL | GGAGCCTCCATATTCTTCAT | Forward |
| CBR610LL | CCTGTTTCGTGGAGAAATAGC | Reverse |
| CBF364LL | GCAACCATAGCAACAGCATT | Forward |
| CBR722LL | GGGGAGAATAGTACTAGC | Reverse |
| CBF500LL | GGTTCTCAGTAGACAAAGCC | Forward |
| CBR1110LL | GCTTGCGATTGGTATGAG | Reverse |
| CBF796LL | CCCCATATCAAACCTGAATGAT | Forward |
| CBR1140LL | GAGTCTTGGGGAGATGGGATTC | Reverse |
