# Supplementary material for: What is the taxonomic status of East Asian otter species based on molecular evidence?: focus on the position of the Japanese otter holotype specimen from museum
Source: Anim Cells Syst (Seoul). 2019 May 2;23(3):228–34. doi: 10.1080/19768354.2019.1601133 (PMC6567078; doi:10.1080/19768354.2019.1601133)
Supplement: Supplemental Material [file TACS_A_1601133_SM5942.docx]

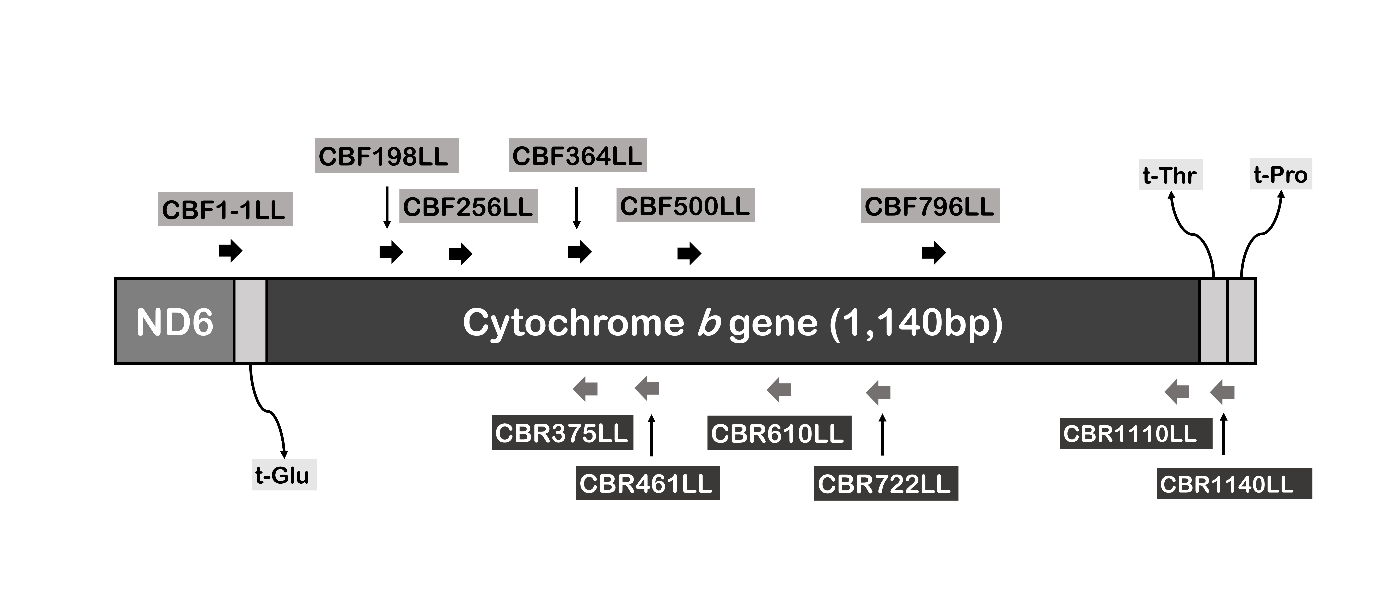


Supplement A. The location of designed primers on cytochrome *b* gene for PCR amplification of ancient DNA of Japanese otter.
